# Zinc Single-Atom Nanozyme As Carbonic Anhydrase Mimic for CO_2_ Capture and Conversion

**DOI:** 10.1021/acsmaterialsau.4c00156

**Published:** 2025-01-31

**Authors:** Eslam M. Hamed, Fun Man Fung, Sam F. Y. Li

**Affiliations:** † Department of Chemistry, National University of Singapore, 3 Science Drive 3, Singapore 117543, Singapore; ‡ Department of Chemistry, Faculty of Science, Ain Shams University, Abbassia, Cairo 11566, Egypt; § School of Chemistry, University College Dublin, Belfield, Dublin 4 D04 C1P1, Ireland

**Keywords:** single-atom, nanozymes, carbonic anhydrase, CO_2_ capture, amino acids

## Abstract

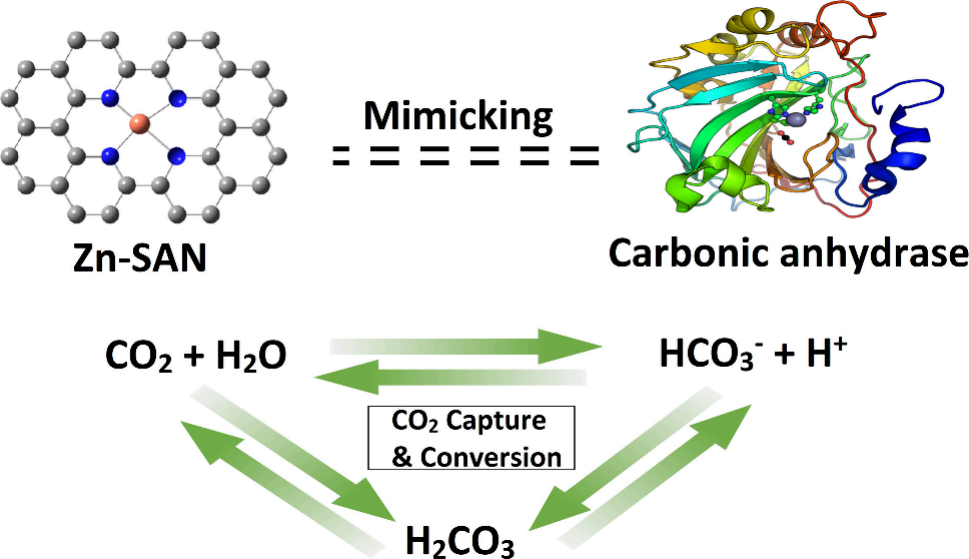

Single-atom nanozymes (SANs) are a class of nanozymes with metal centers that mimic the structure of metalloenzymes. Herein, we report the synthesis of Zn–N–C SAN, which mimics the action of the natural carbonic anhydrase enzyme. The two-step annealing technique led to a metal content of more than 18 wt %. Since the metal centers act as active sites, this high metal loading resulted in superior catalytic activity. Zn-SAN showed a CO_2_ uptake of 2.3 mmol/g and a final conversion of CO_2_ to bicarbonate of more than 91%. CO_2_ was converted via a biomimetic process by allowing its adsorption by the catalyst, followed by the addition of the catalyst to HEPES buffer (pH = 8) to start the CO_2_ conversion into HCO_3_^–^. Afterward, CaCl_2_ was added to form a white CaCO_3_ precipitate, which was then filtered, dried, and weighed. Active carbon and MCM-41 were used as controls under the same reaction conditions. According to the findings, the CO_2_ sequestration capacity was 42 mg of CaCO_3_/mg of Zn-SAN. Some amino acids (AAs) with binding affinity for Zn were able to suppress the enzymatic activity of Zn-SAN by blocking the active metal centers. This strategy was used for the detection of His, Cys, Glu, and Asp with detection limits of 0.011, 0.031, 0.029, and 0.062 μM, respectively, and hence was utilized for quantifying these AAs in commercial dietary supplements.

Serious climate issues are caused by the increase in greenhouse gases (GHGs) such as carbon dioxide (CO_2_) in the atmosphere.^1^ Over the past centuries, there may have been an increase in global temperature due to the release of CO_2_ by human activities.^2^ Therefore, there is an urgent need for efficient ways to capture CO_2_ and reduce its emissions. Several methods, such as chemical sequestration^3^ and physical adsorption,^4^ have been used to lower CO_2_. Nonetheless, many difficulties still exist. For instance, CO_2_ adsorption or sequestration typically requires a substantial investment in equipment regeneration, and commercial methods have the drawbacks of being expensive and producing secondary emissions.

Therefore, the most efficient way to treat CO_2_ may be to capture and convert CO_2_ in situ to other practical products.^5−8^ Carbonic anhydrase (CA), a metalloenzyme with a zinc active site, may hydrate CO_2_ in nature.^9−11^ The twisted Td geometry of Zn centers paired with three nitrogen atoms and one oxygen make up the active site of CA.^12^ A water molecule is crucial for CO_2_ hydrolysis because it provides an OH unit that allows CO_2_ to be converted to bicarbonate ions (HCO_3_^–^).^13,14^

However, the fragile nature of CA, its high cost, and instability against heat and chemicals prevent it from being widely used.^15,16^ The fact that biomimetic catalysts that mimic the CA active site are probably sturdy enough to overcome these issues is one way that nature inspires us. Nanozymes are nanomaterials with inherent enzyme-like properties.^17−23^ Because they may address issues with biological enzymes, including low stability and high cost, they have become increasingly common.^24−31^ Due to their distinct physicochemical properties and catalytic capacities, nanozymes, in particular, have been proven to have a broad variety of applications, from in vitro detection to the replacement of specific enzymes in living systems.^32−36^ Through atomic-level simulation of the highly evolved catalytic core of biological enzymes, single-atom nanozymes (SANs) with unique electrical and geometric configurations may serve as beneficial substitutes for current enzymes.^37−41^

SANs are often criticized for their complex synthesis, limited stability, high costs, and potential toxicity. However, the Zn-SAN developed in this study addresses these limitations effectively. The synthesis process employs a cost-effective and scalable dual-temperature annealing method, resulting in high metal loading (>18 wt %) and efficient active site utilization. Stability tests demonstrate that Zn-SAN remains robust under a wide range of temperatures (up to 500 °C), pH values (2–12), and various organic solvents, ensuring its durability in diverse operational environments. Additionally, zinc is a biologically essential and relatively low-toxicity element compared to other metals commonly used in SANs, making Zn-SAN a safer and more biocompatible option. These features collectively position Zn-SAN as a practical and sustainable alternative to conventional SANs, particularly for catalytic and sensing applications.

Here, a zinc-centered single-atom nanozyme (Zn-SAN) was constructed to mimic the biocatalysis of the CO_2_ transformation property of CA by providing an appropriate chemical environment. A three-coordinate zinc with three nitrogen atoms occupying a distorted trigonal geometry similar to that of natural CA showed that Zn-SAN can efficiently hydrolyze CO_2_ by mimicking its structural and catalytic characteristics, in addition to being able to absorb and store CO_2_. This biomimetic catalyst also has improved stability, is simpler to prepare, and can be recycled. This work opens the door to effective CO_2_ conversion and treatment using biomimetic catalysis.

## Results and Discussion

### Synthesis and Characterization of Zn-SAN

**Figure 1:**
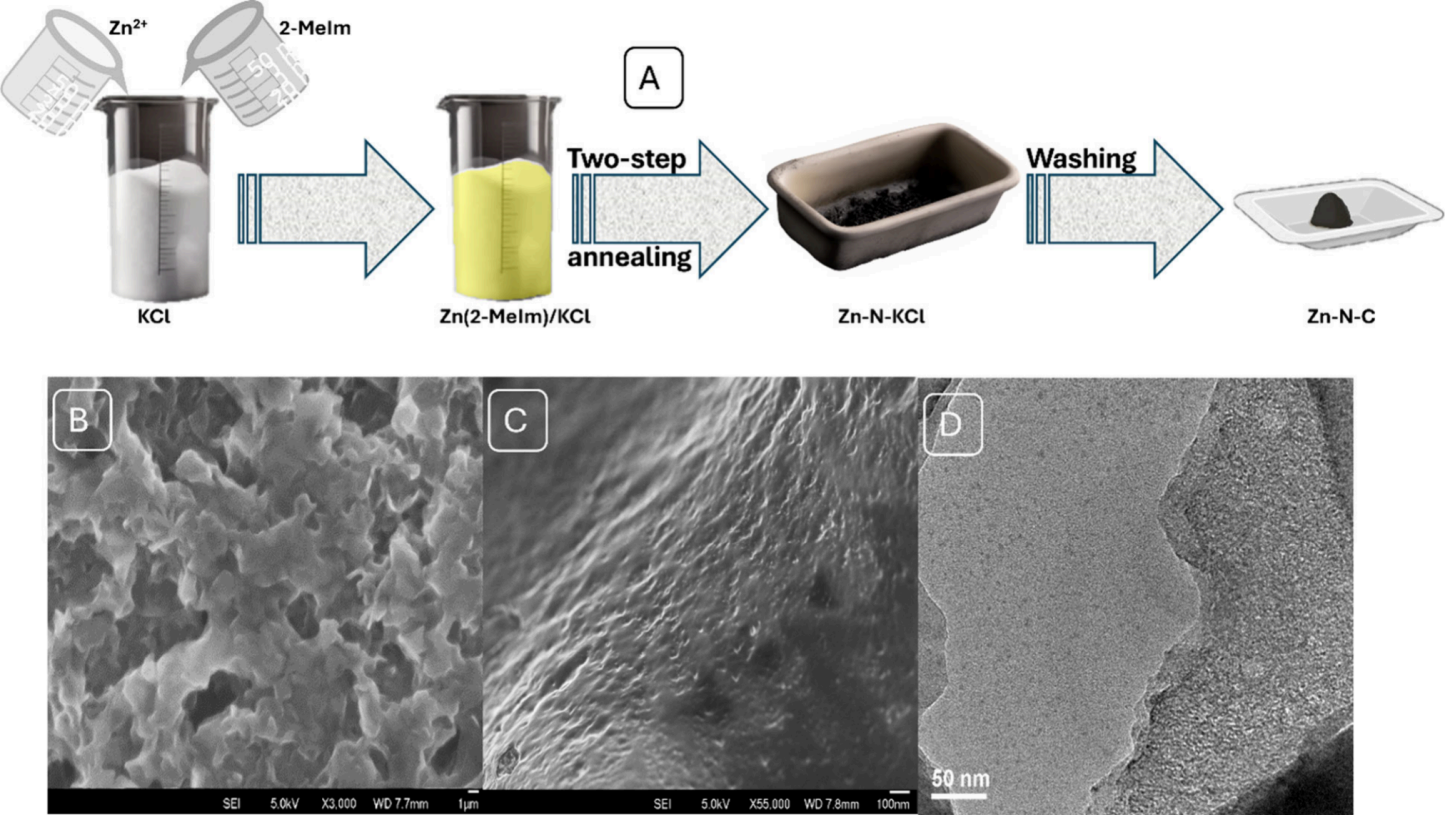
(A) Illustration of the synthesis process of Zn-SAN. (B and C) SEM image showing the 3D network structure of Zn-SAN. Scale bar 1 μm and 100 nm, respectively. (D) TEM image for Zn-SAN. Scale bar 50 nm.

Zn-SAN was synthesized using a previously reported method,^42^ where zinc nitrate was used as the metal precursor, 2-methylimidazole (2-MeIm) was used as the source of carbon and nitrogen, and potassium chloride was used as the solid support. The precursors were mixed with the solid support, and the mixture was pyrolyzed for 5 h at 300 °C with flowing nitrogen and then for 5 more hours at 550 °C. After washing with sulfuric acid and water, the final product was dried overnight at 80 °C. (Figure 1A).

TEM images demonstrated the absence of any nanoparticles (NPs) (Figure S1), and selected-area electron diffraction (SAED) revealed a ring-like pattern and verified the amorphous nature of the SAN (Figure S2) The X-ray diffraction (XRD) pattern provided more evidence by confirming the structure and absence of Zn NPs. This was demonstrated by the presence of two peaks at approximately 27° (002) and 44° (101) for graphitic carbon, but no diffraction peaks for Zn NPs were observed after acid washing (Figure S3). The absence of NPs after acid washing was confirmed by Scanning electron microscopy (SEM) (Figures 1B and 1C) and High-resolution transmission electron microscopy (HRTEM) (Figure 1D).

**Figure 2:**
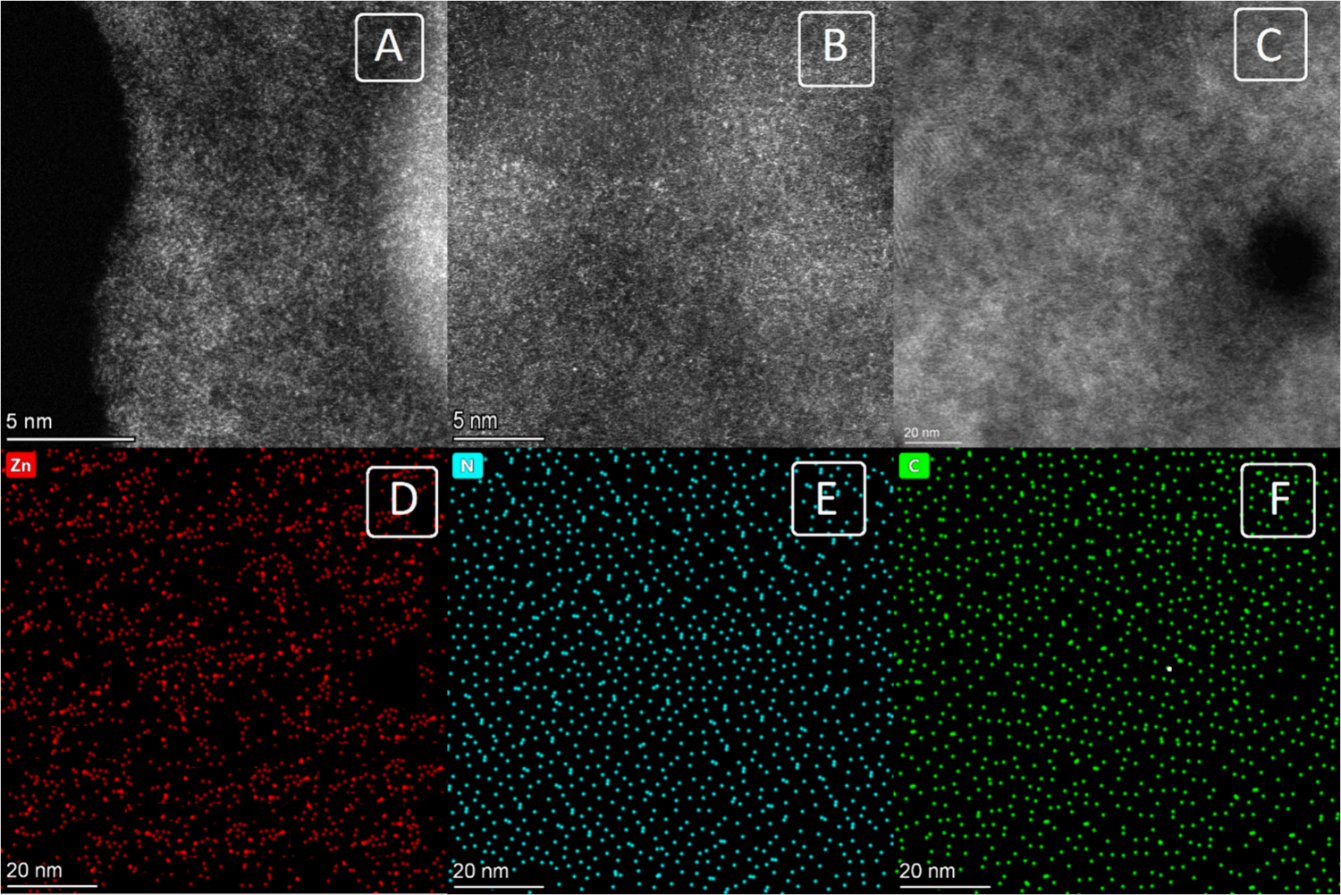
HAADF-STEM image of the Zn-SAN showing the Zn single atoms as the bright dots, scale bar (A and B) 5 nm and (C) 20 nm, and the corresponding EDS elemental mapping showing (D) Zn, (E) N, and (F) C.

The high-angle annular dark field scanning tunneling electron microscopy (HAADF-STEM) results showed that single Zn atoms were evenly distributed throughout the carbon sheet, as depicted in Figures 2A and 2B. Energy dispersive spectroscopy (EDS) mapping demonstrated the uniform distribution of Zn, N, and C inside the SAN structure (Figures 2C–2F). Brunauer–Emmett–Teller (BET) analysis demonstrated that SAN possessed a porous structure with a substantial surface area. The BET surface area of the Zn-SAN material was approximately 1260 m^2^ g^–1^, while the average pore diameter was approximately 1.3 nm, as determined using the Barrett–Joyner–Halenda method. The N_2_ adsorption–desorption isotherms, the corresponding BET surface area, and the micropore-size distribution of Zn-SAN are given in Figure S4. These micropores provide a large surface area per unit mass, which enhances the material’s adsorption capacity. Moreover, the small pore size allows selective adsorption of smaller molecules like CO_2_, making microporous materials highly effective for CO_2_ capture. Additionally, micropores create a confined environment, which can enhance catalytic activity by concentrating reactants at active sites. These micropores are beneficial for the specific purpose of CO_2_ adsorption and conversion, as CO_2_ molecules are small and fit well within micropores.

**Figure 3:**
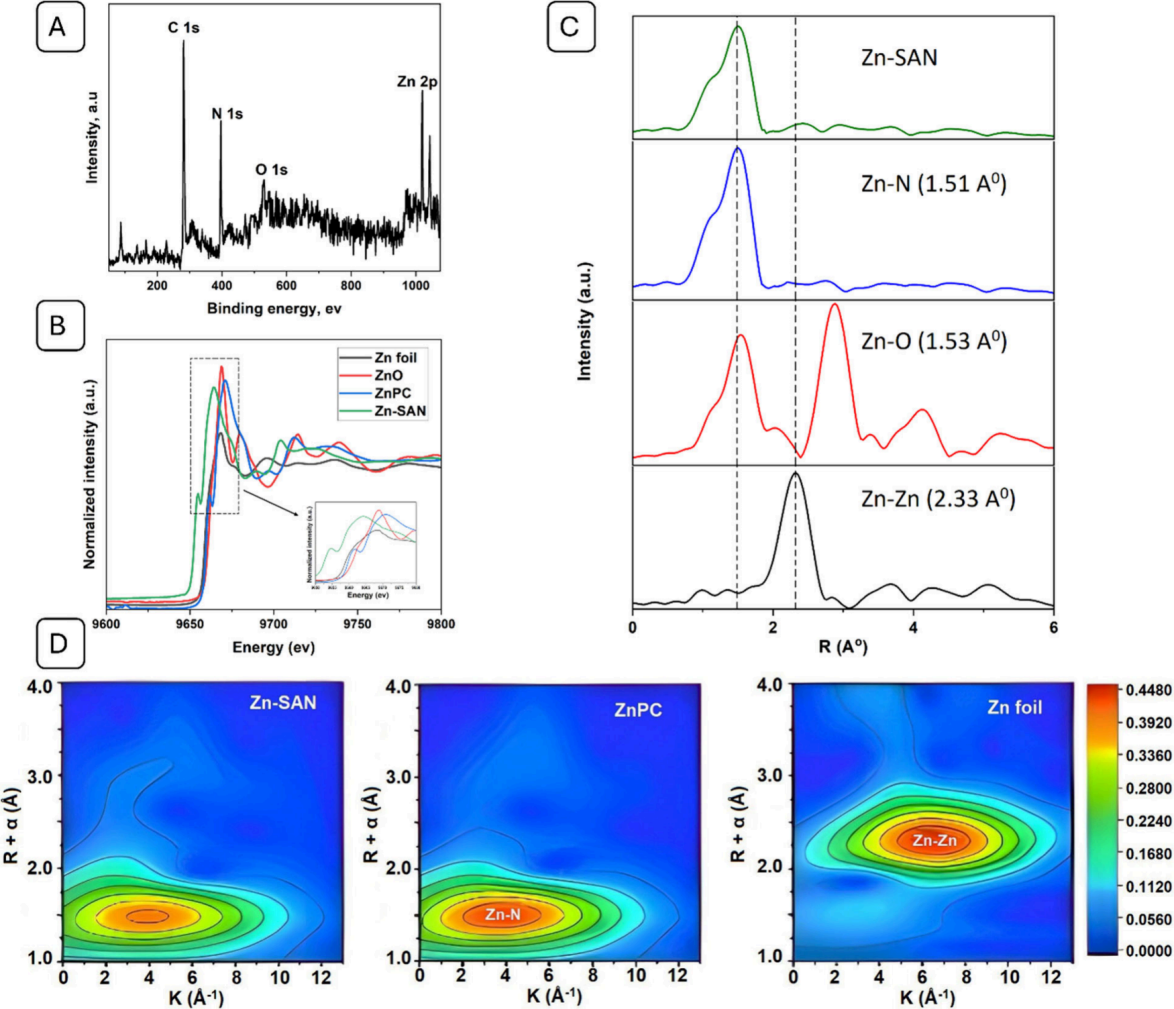
Atomic structure analysis of Zn-SAN. (A) XPS survey scan and (B) Zn K-edge XANES spectra with reference to Zn-foil, ZnO, and ZnPC. Inset is the enlarged view of the peaks at the region of interest. (C) FT-EXAFS spectra at the R-space. (D) The wavelet-transformed EXAFS contour plots of Zn-SAN, ZnPC, and Zn foil.

The binding states of carbon (C) and nitrogen (N) on SAN were analyzed using X-ray Photoelectron Spectroscopy (XPS). The C 1 s spectra agreed well with the peaks observed at 287.6 eV (C–O), 285.2 eV (C–N), and 283.8 eV (C–C) (Figure S5A). The N 1S spectra revealed the presence of graphitic nitrogen (401.3 eV), pyrrolic nitrogen (400.5 eV), zinc–nitrogen (399.8 eV), and pyridine nitrogen (398.6 eV) (Figure S5B). The Zn 2P spectra displayed two strong peaks at 1042.6 and 1019.9 eV for Zn 2*P*_1/2_ and Zn 2P_3/2_, respectively, showing that Zn^1^ species are abundant in Zn-SAN (Figure S5C). Consistent with the XPS results, the elemental compositions of Zn, N, and O were calculated to be 18.74, 21.55, and 2.67%, respectively (Figure 3A). These results showed a very high metal loading in the SAN and that N species were doped into the 3D structure of the carbon sheet.

### Atomic Structure Analysis

The coordination environment of Zn-SAN was examined using extended X-ray absorption fine structure spectroscopy (EXAFS). The magnitudes of the Zn K-edge EXAFS peak after Fourier transformation are displayed (Figure 3C). Zn-SAN exhibited a prominent peak at 1.51 Å, which aligned perfectly with the Zn–N peak observed for zinc phthalocyanine. This peak is ascribed to the backscattering of Zn and N atoms.^43^ The small peak of Zn–Zn observed at 2.34 Å in the Zn-SAN spectrum was far more prominent in the Zn-foil spectrum, suggesting that the majority of the metal present was in the form of individual atoms.^44^

Next, quantitative EXAFS curve-fitting analysis was used to study the coordination setup. The structural characteristics and quantitative chemical configuration of the Zn atoms were determined by fitting the EXAFS at the Zn K-edge (Table S1). It was found that the metal center’s coordination number was approximately 4 based on the quantitative fitting study. The X-ray absorption near-edge structure (XANES) analysis of Zn-SAN provides critical insights into the coordination environment around the zinc centers. The Zn K-edge spectra of Zn-SAN were compared with those of reference materials, including Zn foil, ZnO, and zinc phthalocyanine (Figure 3B). Electron transport between Zn and N caused Zn-SAN to peak at 9,664 eV, which is different from Zn–O and Zn–N at 9,668 eV and 9,671 eV, respectively. A higher peak intensity indicates more electron transport between the coordination atoms and the metal center. Furthermore, the absence of peaks corresponding to metallic Zn or ZnO confirms that the Zn is present exclusively as single atoms, avoiding nanoparticle aggregation. The white-line intensity, associated with the 1s → 4p transition, is significantly lower in Zn-SAN compared to Zn phthalocyanine, suggesting enhanced electron density around the Zn centers due to the nitrogen-rich coordination. This electron enrichment is crucial for the catalytic activity of Zn-SAN as it facilitates the adsorption and activation of CO_2_ molecules. Furthermore, the wavelet-transformed (WT) contour plots of Zn-SAN, ZnPC, and Zn foil are shown in Figure 3D. The intensity of the dominant peak around 1.5 Å in R space and 3.94 Å^–1^ in k space from the WT contour plot of Zn-SAN was similar to that of ZnPC, indicating the presence of a Zn–N bond. In contrast, the prominent peak around 2.33 Å in R space and 6.78 Å^–1^ in k space from the WT contour plot of Zn foil corresponded to the Zn–Zn bond, which was absent in Zn-SAN. This suggests that Zn-SAN contains Zn–N bonding sites, while the Zn–Zn bond is not present. Figure S6 displays the Feffit results for a simple 1-shell fit to a spectrum from Zn SAN in the K- and R-spaces.

### Biomimetic Catalysis and Conversion

To assess the biomimetic activities of Zn-SAN, the hydrolysis of para-nitrophenyl acetate (p-NPA) was first conducted as a CA mimicking reaction. By observing the production of para-nitrophenol (p-NP), at 402 nm, at ambient temperature, the catalytic activity of Zn-SAN was assessed (Figure S7). The results indicate a high activity toward p-NPA compared to that of the Zn ion blank, which showed minimal activity. The catalytic rate of Zn-SAN was found to be 2.86 μM/min. Additionally, the 2 h conversion of p-NPA for Zn-SAN was 47.4%.

**Figure 4:**
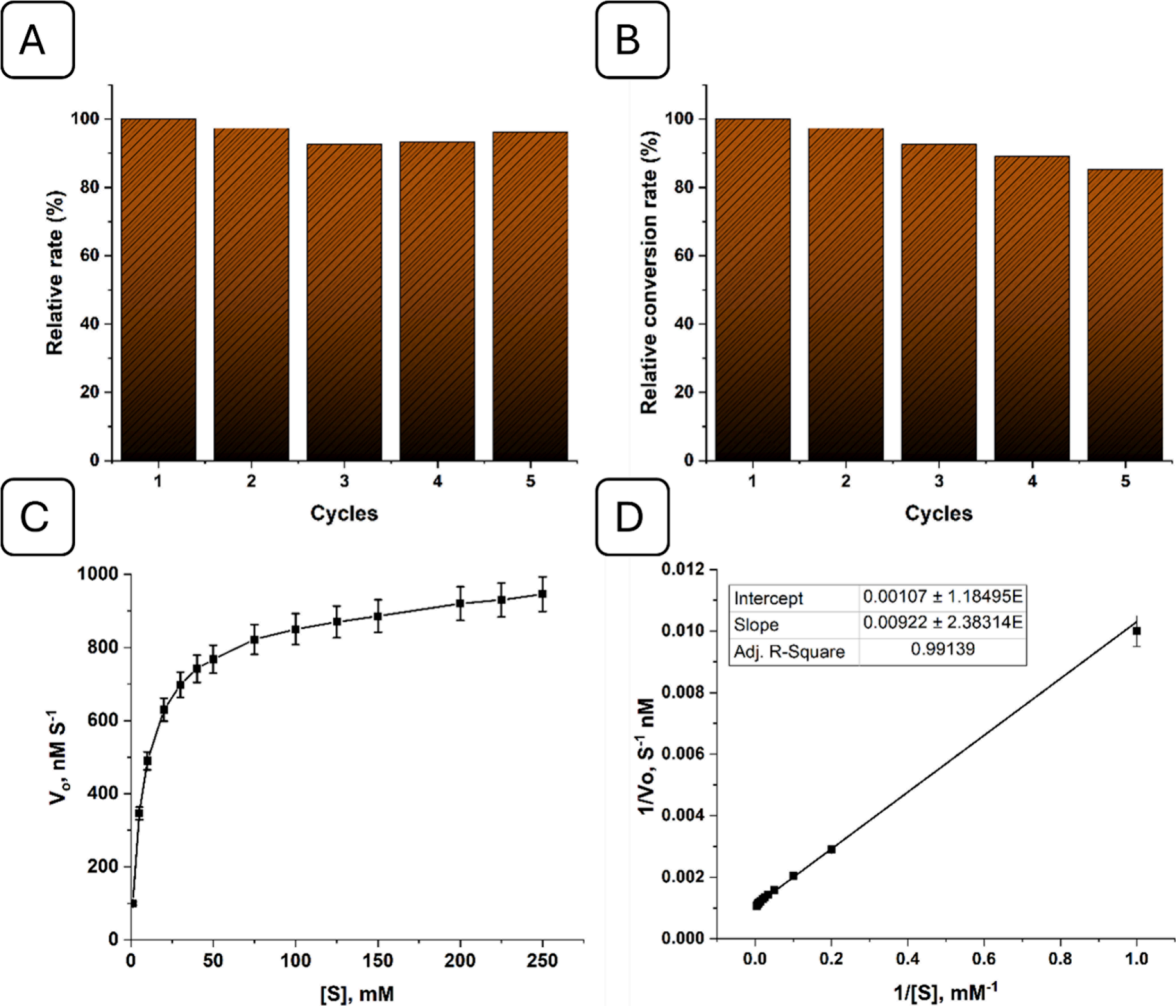
(A) Relative reaction rate of Zn-SAN at multiple sets, (B) relative biomimetic conversion at multiple sets, (C) changes of the initial hydrolysis rate (*V*_0_) of p-NPA catalyzed by Zn-SAN with substrate concentration, and (D) Lineweaver–Burk plots for estimating *V*_max_ and *K*_m_ of Zn-SAN.

Additionally, the reusability of Zn-SAN was assessed for a maximum of five catalytic cycles. Figures 4A and 4B show that Zn-SAN can be recycled and reused five times without causing an apparent decrease in the substrate conversion or reaction rate. Regeneration was carried out under flowing nitrogen gas at 60 °C maintaining a rate of 400 mL/min.

The maximum velocity (Vmax) and Michaelis–Menten constant (Km) of Zn-SAN toward the hydrolysis of p-NPA, were estimated using the correlation between the velocity (V) and the substrate concentration ([S]) from the Lineweaver–Burk plot, where the intercept of the line represents 1/Vmax, while Km is calculated as (Slope x Vmax). The Vmax value for Zn-SAN was 934.6 nM/s and Km was 0.86 mM (Figures 4C and 4D), presenting an outstanding performance compared to all the recent methods in the literature (Table S2).

Zn-SAN’s catalytic activity was assessed in relation to temperature, pH, and solvent stability in order to assess the biomimetic catalyst’s suitability for use in hostile environments and possible industrial uses. Zn-SAN exhibited reasonable stability up to 500 °C, thereby offering the potential to catalyze reactions in high-temperature environments. Additionally, Zn-SAN was stable in a range of solvents (DMF, THF, acetone, and alcohols) and a wide range of pH values (2–12) (Figure S8).

**Figure 5:**
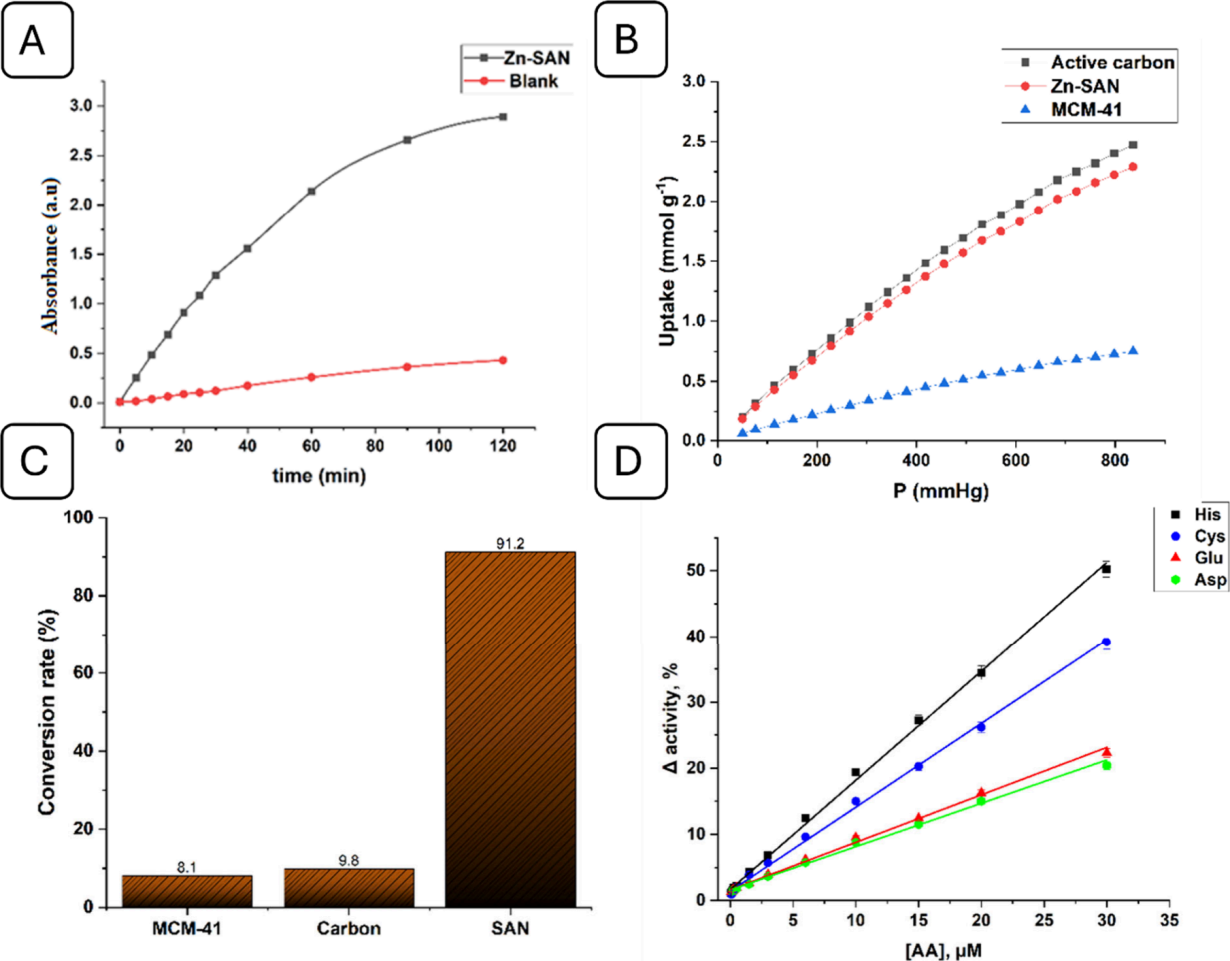
(A) Zn-SAN-based kinetic traces of p-NPA biomimetic catalysis, (B) CO_2_ adsorption isotherms of Zn-SAN compared to carbon and MCM-41 at 25 °C, (C) conversion rate of CO_2_ using Zn-SAN, active carbon, and MCM-41, and (D) calibration plot for the determination of His, Cys, Glu, and Asp using Zn-SAN.

To determine how much CO_2_ the SAN had absorbed, CO_2_ sorption data were initially gathered for this investigation. MCM-41 and active carbon were used as control materials for CO_2_ absorption showing a CO_2_ uptake of 0.7 and 2.5 mmol/g at 25 °C and 1 atm, respectively. However, Zn-SAN showed a CO_2_ uptake of 2.3 mmol/g under the same conditions (Figures 5A and 5B). It is obvious that active carbon showed better CO_2_ uptake than Zn-SAN and MCM-41. Nonetheless, the final CO_2_ conversion for active carbon was less than 10%, while the final conversion of CO_2_ catalyzed by Zn-SAN was more than 91% (Figure 5C). Furthermore, the turnover number (TON) of Zn-SAN was 0.43 mmol/mmol, indicating the high activity site density of the catalyst. From the results, it can be concluded that the proposed Zn-SAN possessed an excellent CO_2_ uptake and extraordinary conversion rate, compared to other methods in the literature (Table S3).

### Amino Acid Analysis

The majority of AAs are small aliphatic molecules that do not glow or absorb UV light, making their analysis difficult.^45^ Numerous methods, including chromatography,^46^ electrochemical assays,^47^ fluorescence,^48^ and colorimetric assays,^49^ have been developed for the detection of AAs. NMR and other cutting-edge methods, such as CE, have also been employed. These methods take considerable time, involve highly skilled operators, and are highly sophisticated.

It was postulated that if an AA has the ability to attach to a Zn metal, it will impede the enzymatic activity of Zn-SAN because AAs can bond with metal atoms and block the active metal centers. With a low detection limit of 0.011 μM for His, 0.031 μM for Cys, 0.029 μM for Glu, and 0.062 μM for Asp, the approach demonstrated a high level of sensitivity (Figure 5D). Selectivity investigations on 20 AAs (GSH, Cys, His, Asp, Glu, Arg, Pro, Gly, Lyc, Tyr, Val, Trp, Ser, Phe, Met, Leu, Ile, Gln, Asp, and Ala) revealed no significant interference with the measurement of His, Cys, Glu, or Asp from any other AA, even at concentrations up to ten times greater (Figure S9).

After the necessary treatment, dietary supplements containing AAs were examined to determine the precise amount of AAs present. Since the AA content of these supplements is significantly greater than the linear range of the suggested method, an appropriate dilution was performed. The results displayed in table S4 indicate that the method recovered a significant percentage of His, whereas Cys, Glu, and Asp showed lower recoveries. It is thought that the performic acid preoxidation step slightly affected the recoveries of these AAs. However, the collected data was still valid and demonstrated the efficacy of the approach.

## Conclusion

In this study, a Zn-centered single-atom nanozyme (Zn-SAN) was engineered to emulate the catalytic properties of the natural carbonic anhydrase enzyme. This innovative design overcomes the limitations of biological enzymes, such as fragility and high cost, by leveraging the unique structural and catalytic properties of single-atom active sites. The Zn-SAN, with its tricoordinated zinc centers and optimized geometry, demonstrates not only exceptional CO_2_ adsorption and conversion efficiency but also remarkable stability under diverse environmental conditions. This work highlights the transformative potential of biomimetic catalysis for addressing critical challenges in greenhouse gas management and sustainable chemical processes. Beyond CO_2_ capture and conversion, the dual-functionality of Zn-SAN for precise amino acid detection underscores its versatility, opening avenues for applications in environmental sensing and industrial catalysis. By offering a robust, scalable, and recyclable solution, this study positions Zn-SAN as a promising platform for future advancements in green chemistry and sustainable technology.

## Methods

### Catalyst Preparation

Zn-NC was synthesized by a one-pot process using zinc nitrate and 2-MeIm as precursors, while potassium chloride served as the solid support. Briefly, 4 mmol of zinc salt was dissolved in 30 mL of methanol (solution A). Separately, 50 mmol of 2-MeIm was dissolved in 30 mL of methanol (solution B). Afterward, the solution (A) was mixed with 500 g of NaCl and left to dry at 80 °C for 30 min. Then, solution (B) was added, and the solution was thoroughly mixed and allowed to dry at 80 °C for 30 min. Subsequently, the salt was submerged in an alumina boat and heated to 300 °C for 5 h at a rate of 3 °C per minute while nitrogen was flowing through a tube furnace. After that, the sample was heated to 550 °C for 5 h while maintaining the same heating rate. After allowing the sample to cool, it was washed several times with DI water and H_2_SO_4_. After drying at 80 °C overnight, SAN was collected for additional use. The salt was then placed in an alumina boat and kept at 300 °C for 5 h under flowing nitrogen in a tube furnace, maintaining a heating rate of 3 °C/min. Subsequently, the temperature was increased at the same heating rate to 550 °C, at which point the sample was kept for 5 h. The sample was then left to cool before multiple steps of washing with DI water and H_2_SO_4_. The SAN was collected for further use after drying at 80 °C overnight.

### Characterization

HRTEM data were recorded using a JEOL JEM-2010F field emission electron microscope, which is a 200 kV class analytical TEM instrument. SEM images were obtained using a JEOL JSM-6701F field emission scanning electron microscope, which was used as a 30 kV class analytical SEM. HAADF-STEM was done using a double spherical aberration electron JEOL JEM-2100F microscope operating at 200 kV. The crystal structure and phase purity were obtained using XRD (Bruker D8 Advance diffractometer with Cu Kα radiation (λ = 1.54056 Å)) at 40 kV and 40 mA. A Kratos Axis Ultra DLD X-ray spectrometer with a monochromatic aluminum Kα X-ray source (energy = 1486.7 eV) was used to perform the XPS measurements. A Cary 60 UV/vis spectrophotometer manufactured by Agilent was utilized for measuring absorbance. To verify the porous structure of the catalyst and determine its surface area, BET analysis was performed at room temperature using a Quantachrome AutoSorb iQ C-XR, which possesses both physisorption and chemisorption capabilities. A 12-h degassing period at 200 °C was followed by adsorption measurements on the sample. X-ray absorption spectroscopy (XAS) measurements were carried out at the Shanghai Synchrotron Radiation Facility (SSRF), located in China, at the BL14W1 station. A 32-element Canberra/XIA Ge solid-state detector and a Si (311) double-crystal monochromator were used to record the spectra in fluorescent mode. Using Demeter Athena and Artemis software, the data were processed in accordance with standard protocols, while the electron storage ring was operated at 3.5 GeV.

### Biomimetic Catalysis and Conversion

The catalytic process was performed at ambient temperature and tracked by monitoring p-NP, a resulting product of the hydrolysis of p-NPA. A series of standard p-NP solutions ranging from 5 to 70 μM were prepared and scanned with a UV/vis spectrophotometer at 402 nm to construct a calibration curve.

The Zn-SAN-catalyzed reactions were carried out in accordance with the recommended procedure to imitate the biocatalysis of CA. p-NPA was initially dissolved in acetonitrile to create a 2.5 mL solution with a concentration of 10 mM. This solution was then made up to 50 mL using HEPES buffer (50 mM, pH = 8), along with a specific quantity of Zn-SAN at a final concentration of 5%. To completely remove the impact of p-NPA’s self-decomposition, the resulting solution was recorded at 402 nm against a blank that was prepared in the same way without the catalyst.^50,51^

Mimicking CA could be used, among other applications, for the capture and conversion of CO_2_, a significant GHG. The biomimetic conversion of CO_2_ was performed by allowing the adsorption of CO_2_ for 2 h at 1 atm with 50.0 mg of the catalyst, followed by the addition of the catalyst to 5.0 mL of HEPES buffer to start the biomimetic CO_2_ conversion. At this stage, the catalyst will catalyze CO_2_ conversion into HCO_3_^–^. After 2 h, 5 mL of 200 mM CaCl_2_ was added to form CaCO_3_ (sup video 1). The white precipitate was then filtered, dried, and weighed. The same reaction conditions were used using active carbon and MCM-41 as a control. The CO_2_ sequestration capacity was found to be 42 mg of CaCO_3_/mg of the Zn-SAN (42 mg/mg) compared to 34.92 mg/mg for the natural CA.

### Amino Acid Analysis

For the detection of AAs, CO_2_ was initially absorbed by 10.0 mg of Zn-SAN for 2 h at 1 atm. and was immersed in 10 mL of HEPES buffer (50 mM, pH = 8) containing different concentrations of His, Cys, Glu, or Asp, maintaining a final concentration of 0.05–30 μM His, 0.1–30 μM Cys, 0.1–30 μM Glu, or 0.2–30 μM Asp. After 2 h of reaction, 5 mL of 500 mM CaCl_2_ was added to the system to form a CaCO_3_ precipitate. Afterward, the precipitate was filtered, dried, and weighed.

Similarly, another solution not containing any AAs was prepared while maintaining the same final volume and concentration. The difference in activity between the samples containing AAs and the corresponding control samples was calculated, and a calibration curve was constructed between the AA concentration and the difference in activity.

## Supporting Information Available

General materials and synthetic methods, experimental protocols, tables and figures, and sample characterization data (PDF)
Video showing the formation of CaCO_3_ upon the addition of CO_2_-loaded Zn-SAN to CaCl_2_ (MP4)
