# Supplementary material for: Zinc Single-Atom Nanozyme As Carbonic Anhydrase Mimic for CO2 Capture and Conversion
Source: ACS Mater Au. 2025 Jan 31;5(2):377–84. doi: 10.1021/acsmaterialsau.4c00156 (PMC11907284; doi:10.1021/acsmaterialsau.4c00156)
Supplement: Supplementary file 1 — mg4c00156_si_001.pdf [file mg4c00156_si_001.pdf]

Zinc single-atom nanozyme as carbonic anhydrase mimic for CO_2 capture and conversion

Eslam M. Hamed[a,b,*], Fun Man Fung[c,*], Sam F. Y. Li[a,*]

[a] Department of Chemistry, National University of Singapore, 3 Science Drive 3, Singapore 117543, Singapore

[b] Department of Chemistry, Faculty of Science, Ain Shams University, Abbassia, Cairo 11566, Egypt

[c] School of Chemistry, University College Dublin, Belfield, Dublin 4, D04 C1P1, Ireland

Table of Contents

Supplementary information.....1
Experimental Procedures.....1
Chemicals.....1
CO_2 absorption calculations.....2
Amino acids supplements treatment.....2
Supplementary figures.....3
Supplementary tables.....12
Supplementary References.....16

Supplementary information

Experimental Procedures

Chemicals

2-methylimidazole, zinc nitrate hexahydrate, sodium chloride, methanol, para-nitrophenol, acetonitrile, HEPES buffer, calcium chloride, amino acids (GSH, Cys, His, Asp, Glu, Arg, Pro, Gly, Lyc, Tyr, Val, Trp, Ser, Phe, Met, Leu, Ile, Gln, Asp, Ala), DMF, THF, acetone, ethanol, and MCM-41 were obtained from *Sigma-Aldrich*.

CO_2 absorption calculations

The CO_2 absorption rate (R_t) was calculated by:

$$R_t = \frac{y_1 - y_{2,t}}{1 - y_{2,t}} x \frac{G_m}{V_L}$$

where y_1 is the mole fraction of CO_2 in the inlet gas (y1 = 0.1); $y_{2,t}$ is the mole fraction of CO_2 in the outlet gas at time t; Gm is the mole flowrate of the inlet gas (Gm = 2.98×10^{-4} mol/s); and V_L is the volume of absorption liquid in the bubble column ($V_L = 3.0 \times 10^{-5}$ m^3).

The amount of CO^2 absorbed (m) over a period was calculated by

$$m = \int_0^t Rt\, dt$$

where T is the absorption time (600 s for round 1, and 275 s for rounds 2–5).

The average CO_2 absorption rate ($\bar{R}$) was calculated by:

$$\bar{R} = \frac{m}{T}$$

Amino acids supplements treatment

Horbäach L-Cysteine (1000mg), *Horbäach* L-Histidine (1000mg), *Healthy Hey* L-Aspartic acid (500mg) and *Swanson* Glutamic acid (500mg) dietary supplement capsules were purchased online from Amazon SG. For all products, the capsules were opened and only the powders were used for analysis. L-Cysteine supplement was pre-oxidized with performic acid before acid hydrolysis with 5 M HCl at 110±3 ^oC for 24 hours[1]. Following the acid hydrolysis, the volume was made up to 100mL and this solution was stored in the fridge at 4^oC. Just before analysis, the working solution was prepared by diluting 1 mL of Asp or Glu, or 0.2 mL of Cys or His stock solutions to 100 mL using DI water.

Supplementary figures

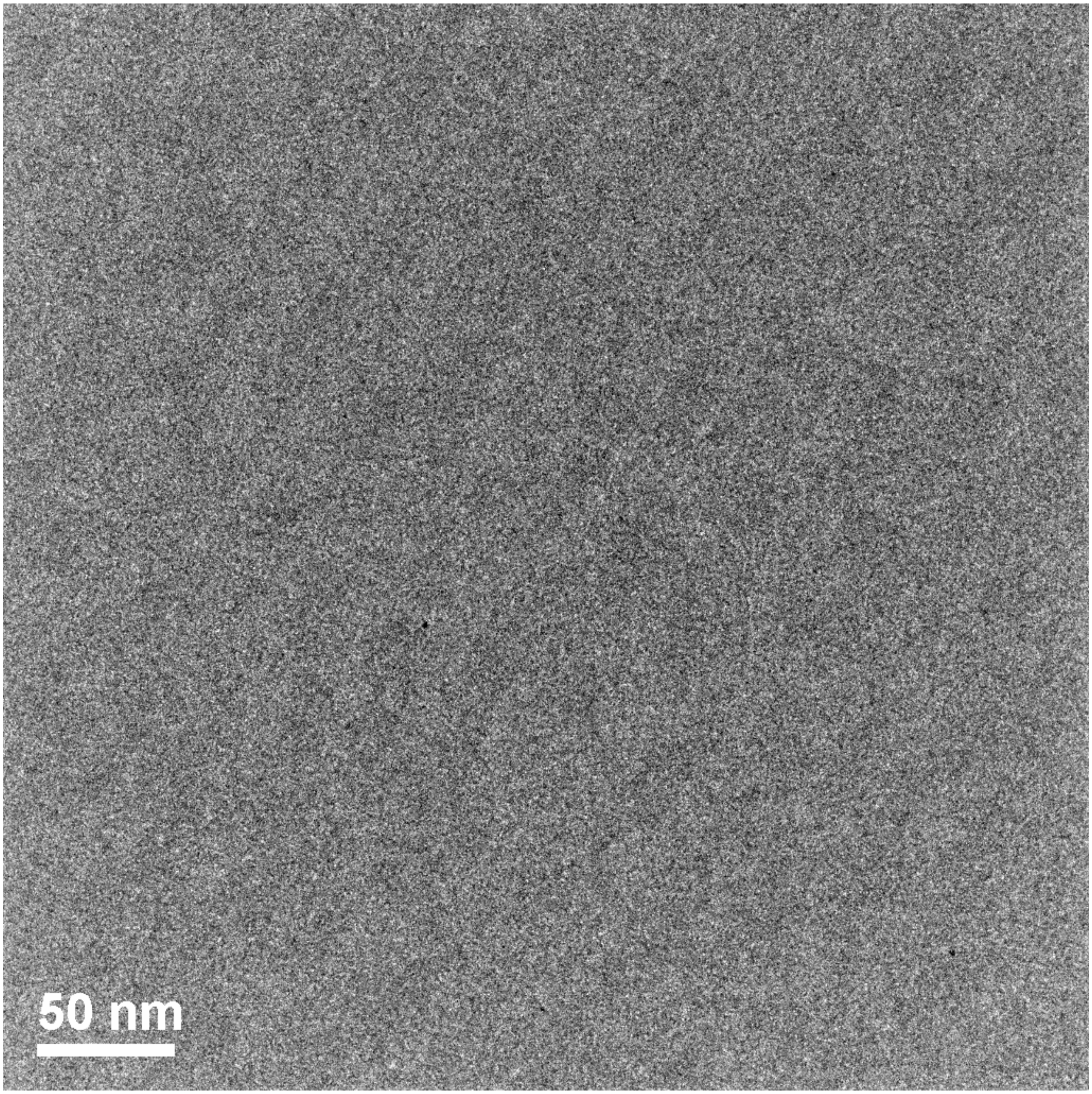

Figure S1. TEM image of Zn-SAN. Scale bar 50 nm.

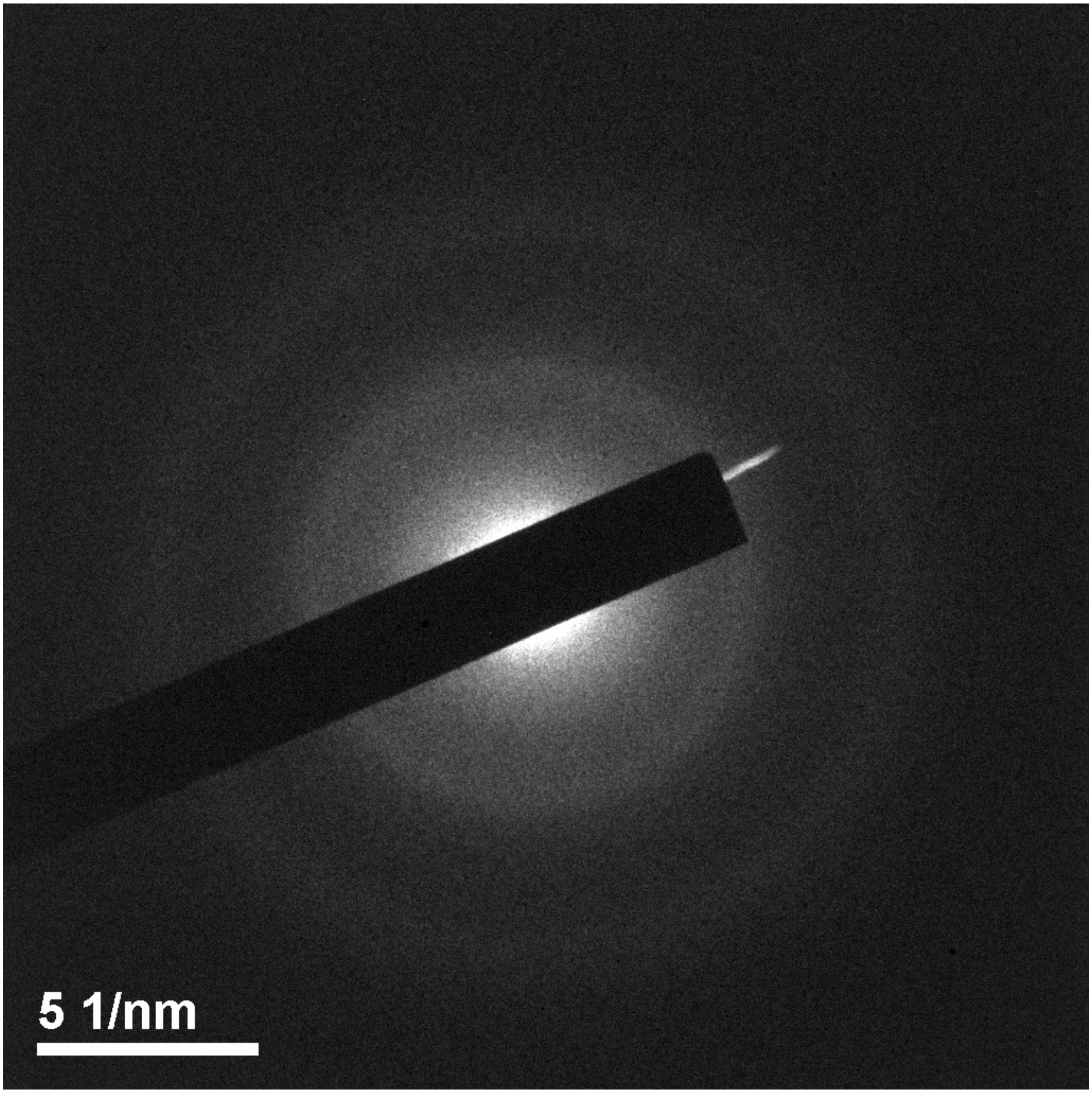

Figure S2 . The SAED pattern of Zn-SAN

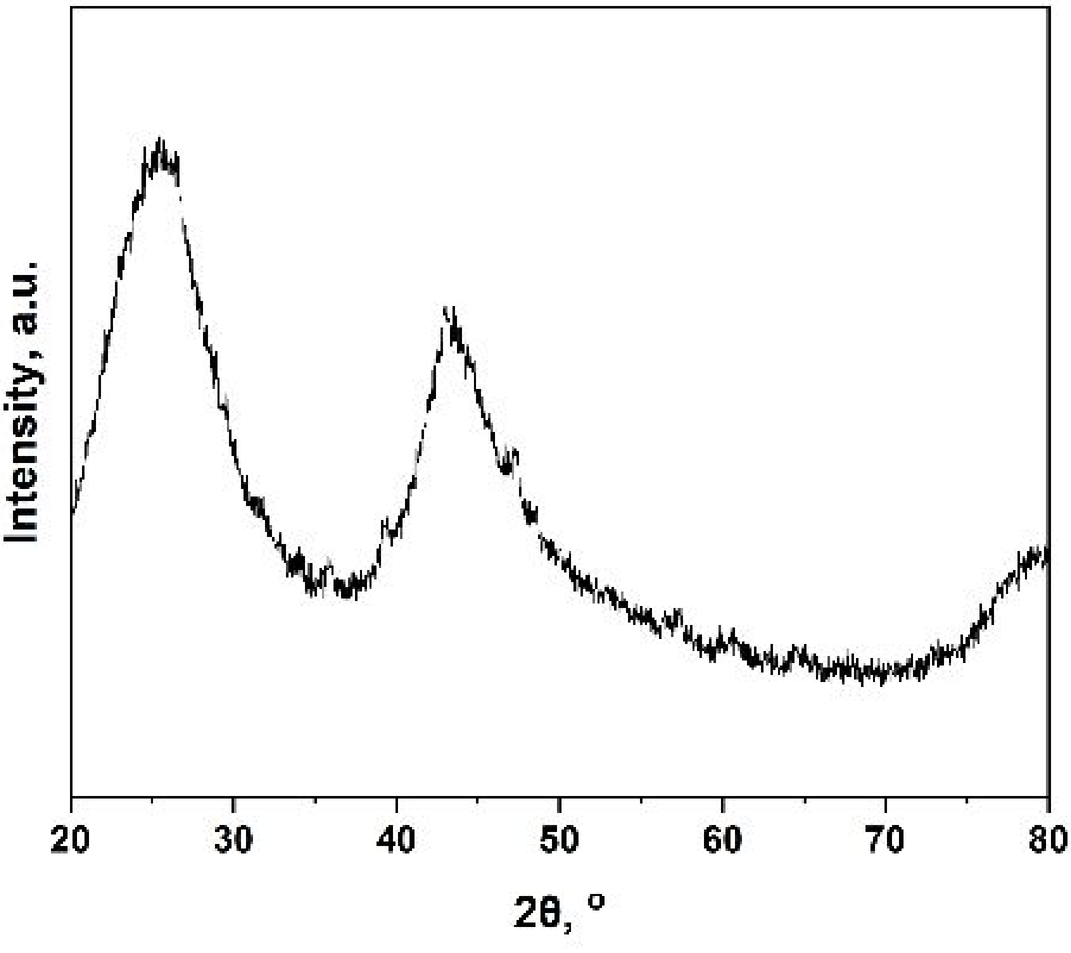

Figure S3 XRD pattern of Zn-SAN showing the two peaks corresponding to the graphitic carbon

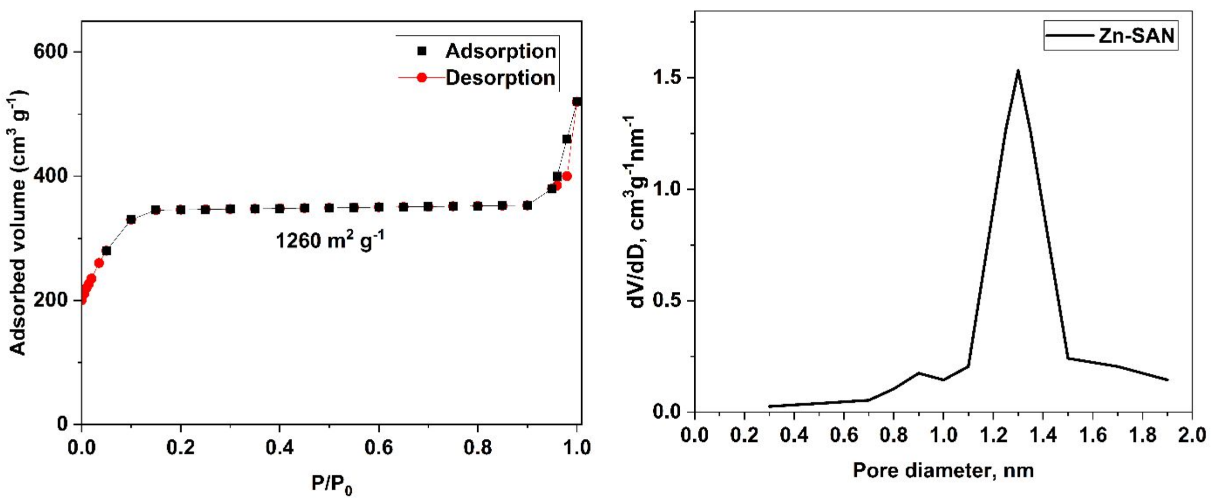

Figure S4 The N_2 adsorption-desorption isotherms, the corresponding BET surface area, and the micropore-size distribution of Zn-SAN

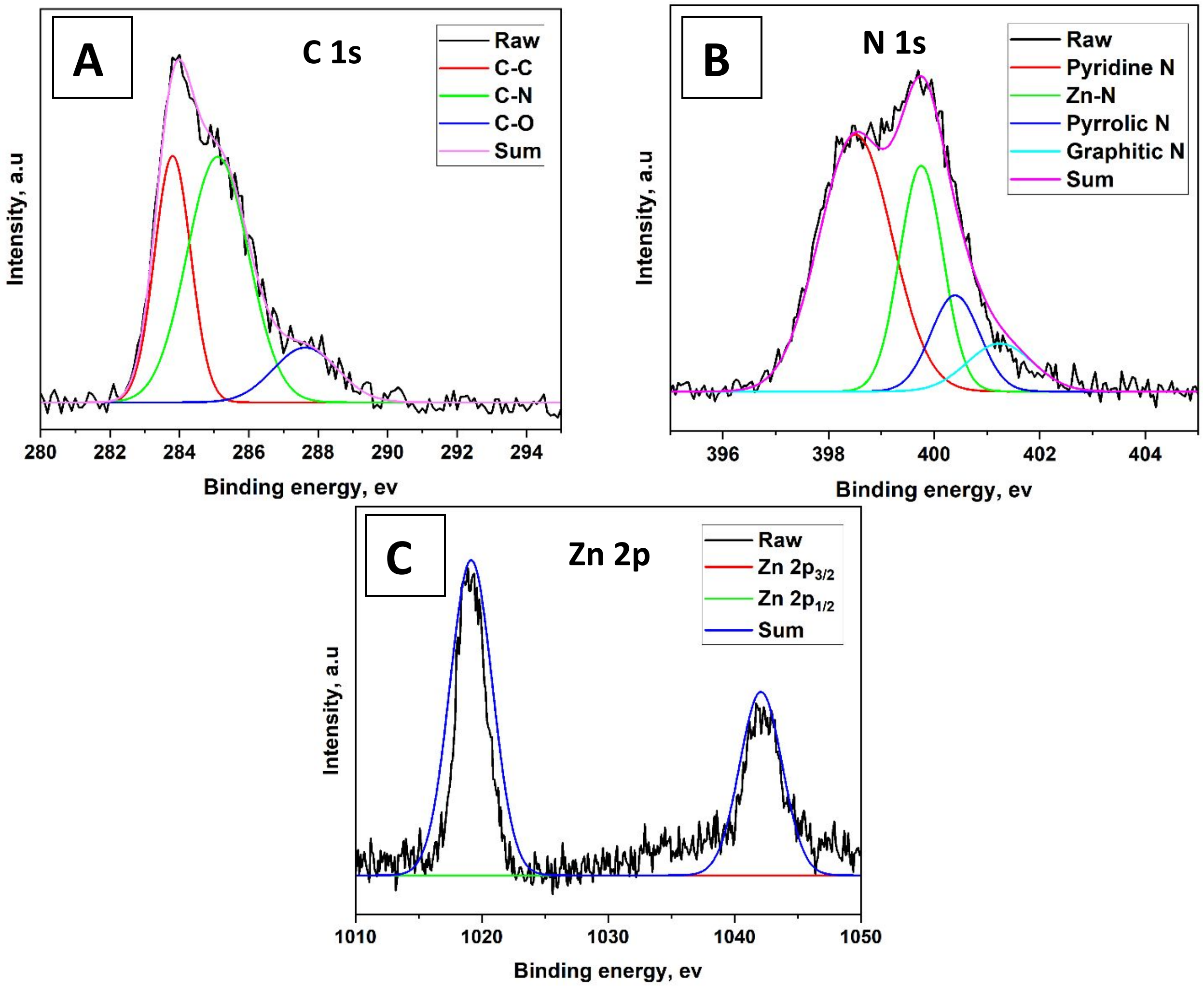

Figure S5. XPS spectra of A) C 1S, B) N 1S, and C) Zn 2P of the Zn-SAN

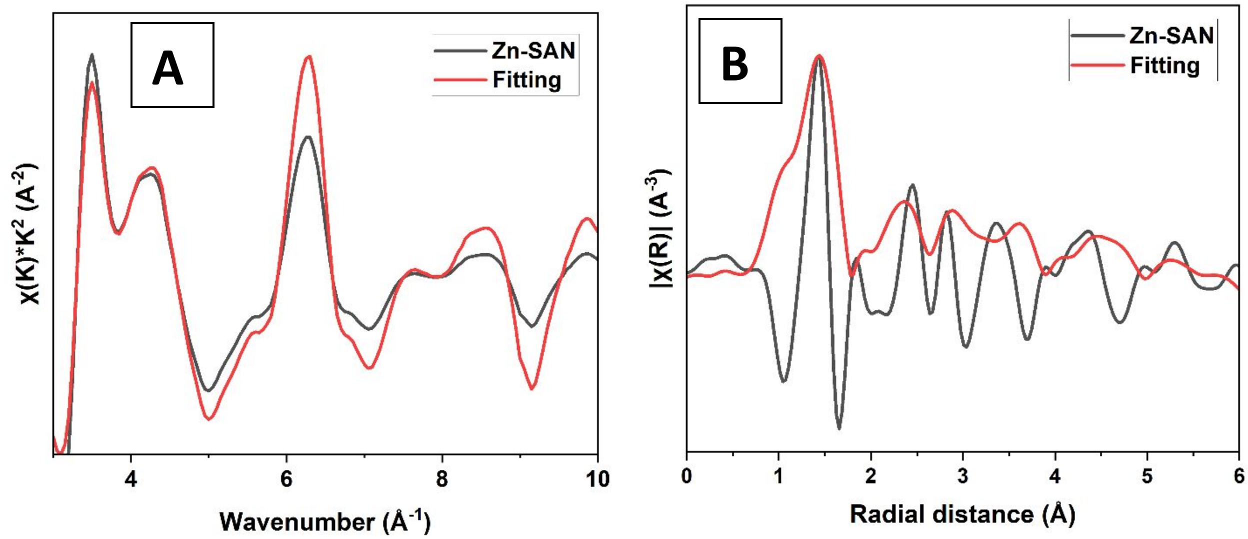

Figure S6. Feffit for the Zn-SAN spectrum in A) K- and B) R-Space

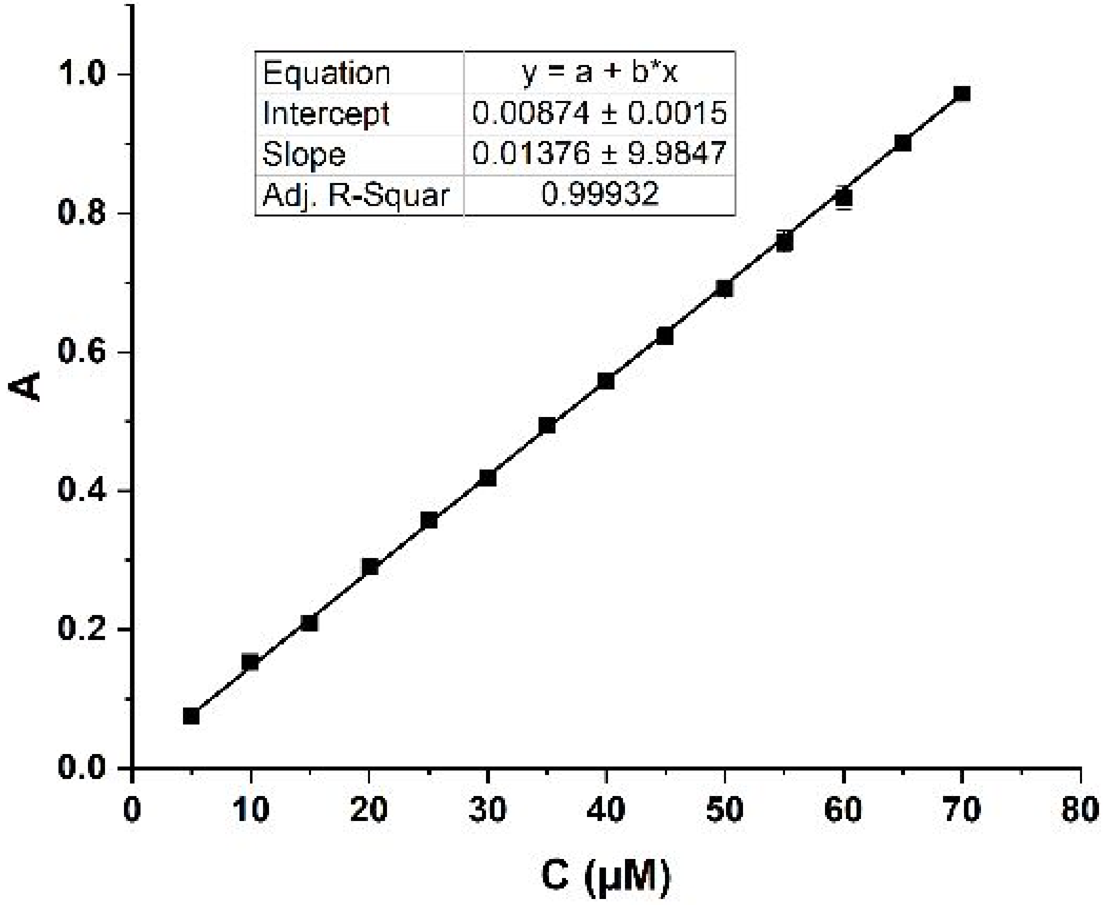

Figure S7. Calibration curve of p-NPA in the range of 5-70 μM

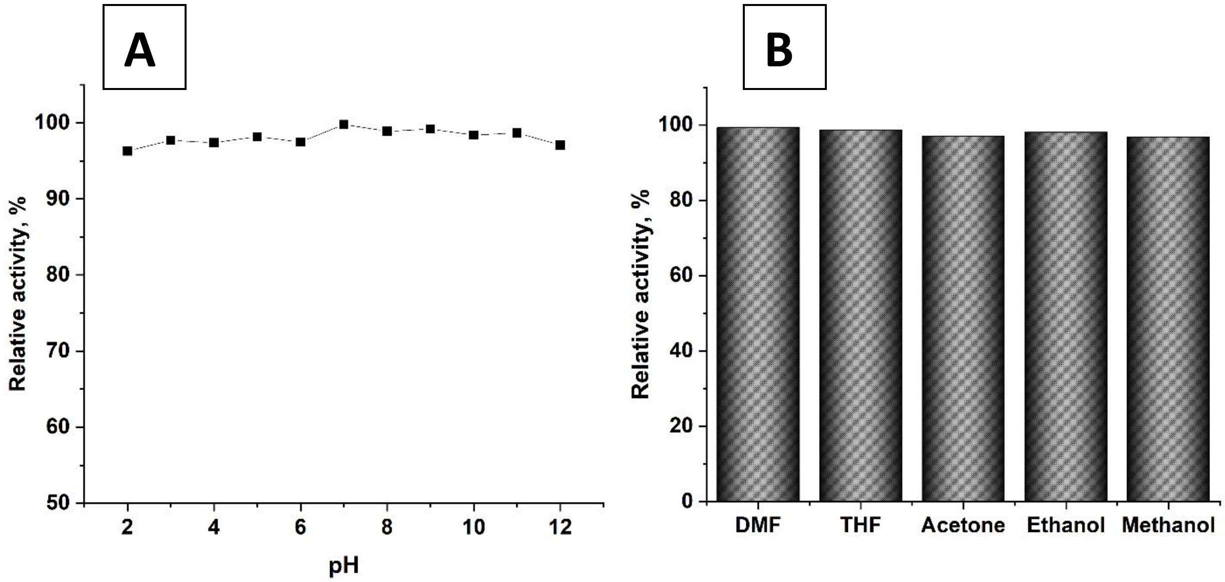

Figure S8. The stability of Zn-SAN under A) a wide range of pH, and B) different solvents

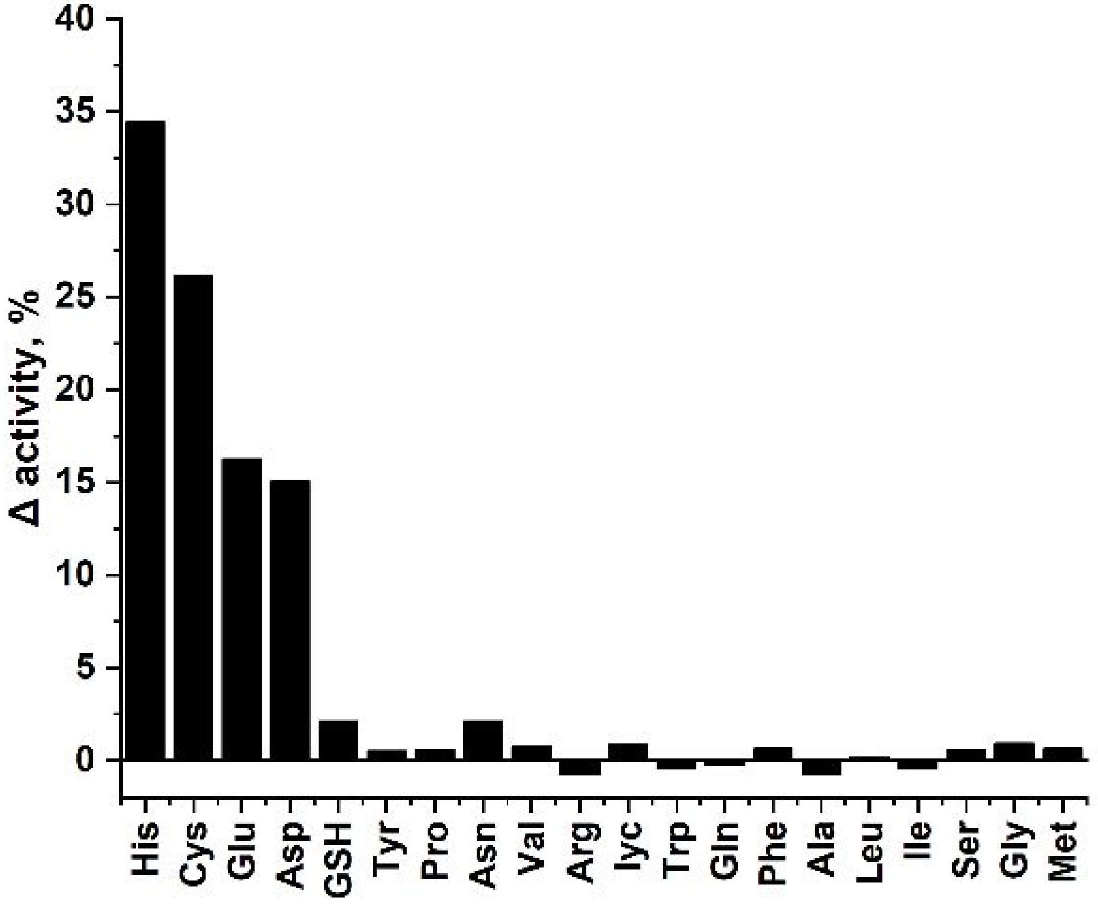

Figure S9 Effect of different amino acids on suppressing the catalytic activity of Zn-SAN

Supplementary tables

Table S1. EXAFS fitting parameters at the Zn K-edge for Zn-SAN

	Bonding	CN	R(A^o)	σ^2(10^{-3}Å^2)	ΔE_0 (eV)	R factor
Zn-SAN	Zn-N	3.6±0.5	1.51±0.02	6.5±0.6	2.36±1.37	0.01
ZnPC	Zn-N	3.9±0.3	1.51±0.01	3.4±0.8	5.34±1.18	0.08
Zn foil	Zn-Zn	12	2.69±0.01	13.8±2.5	-2.6±1.0	0.11

CN: coordination number; R: bond distance between Zn central atoms and surrounding coordination atoms; σ^2: Debye-Waller factor; ΔE_0: inner potential shift; R factor: goodness of fit.

Table S2. Kinetic parameters of p-NPA hydrolysis catalyzed by Zn-SAN compared to CA and other methods in the literature

Catalyst	*V*max (nM/s)	*K*m (mM)	Ref
Zn-N-C SAN	934.6	0.86	This work
CA	54,833	0.24	[2]
Ala–ZIF-8	618.4	13.7	[2]
ZIF-67	633.6	29.2	[3]
$Co_{50\%/}$ZIF-8	461.4	0.82	[3]
Fe_{10}@CN-Mg	512.3	6.37	[4]
ZIF-8	408.8	23.5	[5]
ZIF-8-TA	561.56	0.65	[6]

Table S3. Average CO_2 Absorption Rate, CO_2 Absorption Amount, and enhancement percentage of Zn-SAN compared to other catalysts in the literature

Catalyst	Absorption Rate ($x10^2$ mol m^{-3} s^{-1})	Absorption Amount (mol m^{-3})	Enhancement percentage (%)	Ref
Water	32.65	19.27		
Zn-N-C SAN	62.32	47.02	90.9	This work
ZIF-8-TA	55.88	31.04	81.8	[6]
ZnTMPyP	55.36	66.43	44.25	[7]

Table S4. Results for amino acids analysis in amino acid supplements using the proposed method (n=3).

AA	Spiked (µM)	Found (µM)	Amount in original sample (mg)	% Recovery
His (1000mg)	0	12.81±0.12	990.9±9.8	99.1
	5	17.84±0.23	994.2±11.2	99.4
	10	22.41±0.28	996.1±13.5	99.6
Cys (1000mg)	0	16.83±0.14	961.41±10.1	96.1
	5	21.78±0.22	964.12±14.6	96.4
	10	26.92±0.37	966.71±12.2	96.7
Glu (500mg)	0	13.69±0.11	485.5±5.7	97.1
	5	18.75±0.20	488.4±6.7	97.7
	10	23.83±0.29	492.6±8.9	98.5
Asp (500mg)	0	15.96±0.17	484.3±6.3	96.9
	5	20.93±0.34	489.7±7.9	97.9
	10	26.06±0.36	491.7±8.2	98.3

Supplementary References

(1) Gilani, G. S.; Xiao, C.; Lee, N. Need for accurate and standardized determination of amino acids and bioactive peptides for evaluating protein quality and potential health effects of foods and dietary supplements. *J. AOAC Int.* **2008**, *91* (4), 894-900. From NLM.

(2) Sun, S.; Zhang, Z.; Xiang, Y.; Cao, M.; Yu, D. Amino Acid-Mediated Synthesis of the ZIF-8 Nanozyme That Reproduces Both the Zinc-Coordinated Active Center and Hydrophobic Pocket of Natural Carbonic Anhydrase. *Langmuir* **2022**, *38* (4), 1621-1630. DOI: https://doi.org/10.1021/acs.langmuir.1c03118.

(3) Xiang, Y.; Yu, D.; Qin, C.; Deng, J.; Wang, X.; Ge, B.; Huang, F. Bimetallic zeolitic imidazolate frameworks Co/ZIF-8 crystals as carbonic anhydrase-mimicking nanozyme. *Colloids and Surfaces A: Physicochemical and Engineering Aspects* **2024**, *685*, 133227. DOI: https://doi.org/10.1016/j.colsurfa.2024.133227.

(4) Zhu, F.; Qiu, H.; Wang, F.; Zhang, X.; Lu, G.-P.; Lin, Y.; Huang, H. Bio-Inspired Iron-Based Carbonic Anhydrase Mimic for CO2 Hydration and Conversion. *ACS Sustainable Chemistry & Engineering* **2023**, *11* (19), 7388-7397. DOI: https://doi.org/10.1021/acssuschemeng.3c00209.

(5) Sun, S.; Xiang, Y.; Xu, H.; Cao, M.; Yu, D. Surfactant regulated synthesis of ZIF-8 crystals as carbonic anhydrase-mimicking nanozyme. *Colloids and Surfaces A: Physicochemical and Engineering Aspects* **2022**, *648*, 129103. DOI: https://doi.org/10.1016/j.colsurfa.2022.129103.

(6) Zhou, Y.; Wang, Y.; Chen, Z.; Gong, H.; Chen, L.; Yu, H. Highly Hydrophilic ZIF-8 as a Carbonic Anhydrase Mimetic Catalyst for Promoting CO2 Absorption. *The Journal of Physical Chemistry C* **2023**, *127* (15), 7184-7196. DOI: https://doi.org/10.1021/acs.jpcc.3c00345.

(7) Zhou, Y.; Chen, Z.; Gong, H.; Wang, X.; Chen, L.; Yu, H. Study on the zinc porphyrins as potential carbonic anhydrase mimics for promoting CO2 absorption in K2CO3 solution. *Chemical Engineering Journal* **2024**, *481*, 148690. DOI: https://doi.org/10.1016/j.cej.2024.148690.
